# Computed tomography and coronary artery calcium score for screening of coronary artery disease and cardiovascular risk management in asymptomatic individuals

**DOI:** 10.1007/s12471-024-01897-1

**Published:** 2024-10-02

**Authors:** Sara-Joan Pinto-Sietsma, Birgitta K. Velthuis, Nick S. Nurmohamed, Rozemarijn Vliegenthart, Fabrice M. A. C. Martens

**Affiliations:** 1 Department of Epidemiology and Data Science, Amsterdam University Medical Center, Amsterdam, The Netherlands; 2 Department of Vascular Medicine, Amsterdam University Medical Center, Amsterdam, The Netherlands; 3 Department of Radiology, University Medical Center Utrecht, Utrecht, The Netherlands; 4 Department of Radiology, University Medical Center Groningen, Groningen, The Netherlands; 5 Department of Cardiology, Amsterdam University Medical Center, Amsterdam, The Netherlands

**Keywords:** Coronary calcium scoring, (Non)-contrast coronary CT scan, Atherosclerosis cardiovascular disease, Risk prediction

## Abstract

Several risk prediction models exist to predict atherosclerotic cardiovascular disease in asymptomatic individuals, but systematic reviews have generally found these models to be of limited utility. The coronary artery calcium score (CACS) offers an improvement in risk prediction, yet its role remains contentious. Notably, its negative predictive value has a high ability to rule out clinically relevant atherosclerotic cardiovascular disease. Nonetheless, CACS 0 does not permanently reclassify to a lower cardiovascular risk and periodic reassessment every 5 to 10 years remains necessary. Conversely, elevated CACS (> 100 or > 75th percentile adjusted for age, sex and ethnicity) can reclassify intermediate-risk individuals to a high risk, benefiting from preventive medication. The forthcoming update to the Dutch cardiovascular risk management guideline intends to re-position CACS for cardiovascular risk assessment as such in asymptomatic individuals. Beyond CACS as a single number, several guidelines recommend coronary CT angiography (CCTA), which provides additional information about luminal stenosis and (high-risk) plaque composition, as the first choice of test in symptomatic patients and high-risk patients. Ongoing randomised studies will have to determine the value of atherosclerosis evaluation with CCTA for primary prevention in asymptomatic individuals.

## Introduction

Various risk prediction models are used to predict atherosclerotic cardiovascular disease (ASCVD) in asymptomatic populations. Prevention of ASCVD requires timely and precise identification of individuals at increased risk so that lifestyle or drug interventions can be recommended. Unfortunately, large systematic reviews only show limited usefulness of most risk prediction models, since they are often not externally validated and there is substantial heterogeneity among predictors and outcome [1, 2]. More importantly, risk prediction models *as a whole* overestimate risk [3]. This has been extensively demonstrated by several large prospective cohort studies, with follow-up periods of 10 years, showing that overestimation of current risk prediction models is more common than underestimation [3, 4], although underestimation is of importance in younger age groups, individuals of low social economic background or certain ethnic groups. One way to address overestimation is by updating the current risk prediction models, such as SCORE2 [5]. On the other hand, one might also improve risk prediction by adding new markers to existing models, such as the coronary artery calcium score (CACS) derived from non-contrast cardiac CT.

CACS gives an estimate of the underlying coronary atherosclerosis, which reflects the impact of all risk factors, known and unknown—and the vulnerability of the individual to these risk factors—on the arterial wall [6]. As such, CACS may give an improved estimate of ASCVD risk [7]. However, the position of CACS for risk prediction is still debated. Important issues discussed below are the positive and negative predictive values; how to position CACS within the current risk prediction models for the asymptomatic individual or as screening tool for the population; its cost-effectiveness; issues regarding therapy; and the potential future role of coronary CT angiography (CCTA) in asymptomatic individuals.

## Current role of CACS in predicting CV disease

### Positive predictive value: adding CACS to current cardiovascular disease risk prediction models

CACS provides incremental value above and beyond current ASCVD risk prediction models, as was concluded in a 2022 meta-analysis of 6 prospective cohort studies comprising almost 18,000 individuals followed for almost 10 years [8]. In addition, McClelland et al. investigated and externally validated a new risk prediction model including CACS [9]. They showed, in two independent validation cohorts, a significant improvement in risk prediction (C-statistics) from 0.7 to around 0.8, when using the new risk equation including CACS. Although the net reclassification improvement (NRI) is not the best way to analyse the incremental value of a marker [10], several studies showed a substantial category-based NRI of 14–32% when adding CACS to different risk prediction models [11–13]. A recent prospective study showed the discriminatory power of CACS beyond SCORE2, which is the risk scoring algorithm included in the most recent ESC guideline: C index for cardiovascular events was 0.61 for SCORE2, and 0.75 when CACS was added [14]. Therefore, there is an abundance of evidence that CACS improves the risk prediction of cardiovascular disease in primary prevention among asymptomatic individuals.

### Negative predictive value: the role of CACS in ruling out significant CAD (‘the power of zero’)

Continuing evidence shows that the *absence* of coronary calcification (CACS 0) in asymptomatic individuals is related to a very low risk of ASCVD, even in individuals with a high risk according to the currently used conventional risk prediction models. A systematic review of 13 studies comprising 64,873 asymptomatic individuals, followed for around 4 years, showed that 40% had a CACS 0, and that the 10-year CVD risk of these individuals was only 0.56% [15]. Two other independent cohorts, comprising 16,529 asymptomatic individuals followed for a longer period of 10 [16] or 15 [17] years, both showed that in individuals with CACS 0, the 10-year CVD risk was on average only 3.2%. In all studies the low 10-year ASCVD risk was independent of the presence of traditional risk factors. In fact, in one of these studies, asymptomatic individuals with CACS 0 had an even lower 10-year CVD risk (2.8%) than individuals who were categorised in the low-risk group (meaning absence of traditional risk factors), assessed by current risk models (3.3%) [16]. In addition, in one of the largest cohorts, comprising 44,052 asymptomatic individuals followed for an average of 6 years, individuals with more than three risk factors (e.g. diabetes, dyslipidaemia, hypertension, smoking) and CACS 0 had a 10-year ASCVD mortality risk of only 2% [18]. This indicates that even in asymptomatic individuals classified as (very) high risk because of the presence of multiple risk factors [16, 17, 19], type 2 diabetes [19] or familial hypercholesterolaemia [20], the absence of coronary calcium is associated with an extremely low ASCVD event rate.

### Limitations of CACS

There are some limitations to consider when using cardiac CT for cardiovascular risk prediction, such as radiation exposure, healthcare costs and incidental non-cardiac findings. All modern scanners can now acquire a cardiac CT for CACS with radiation doses < 1 mSv [21], a radiation exposure that is much lower than the annual background radiation. The cost of a non-contrast cardiac CT also depends on the time required for calculating the CACS and assessing the scan for incidental findings. Costs can be reduced by automatically calculating CACS with the current software and omitting assessment for incidental findings. On the other hand, one could argue that relevant incidental non-cardiac findings are not a costly burden but a blessing in disguise, such as early lung carcinoma detection. Further studies are needed to adequately assess cost-effectiveness, taking into account incidental non-cardiac findings. Finally, CACS cannot rule out the presence of non-calcified atherosclerotic plaque and possible high-risk plaque features. However, all studies with long-term follow-up show that the negative predictive value of CACS 0 for cardiovascular events is excellent in asymptomatic individuals. Furthermore, Mortensen et al. showed in 23,143 individuals who underwent CACS and CCTA that a CACS of 0 resulted in a very low event rate of 6.9 per 1000 person-years, regardless of whether there was no plaque, purely non-calcified plaque or even non-calcified plaque with > 50% stenosis [22].

### Positioning of CACS in the Dutch guideline on CVRM

**Fig. 1:**
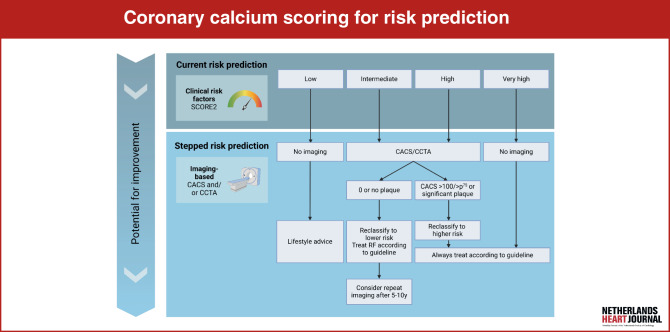
Infographic: Stepped approach with coronary calcium score. *CACS* coronary artery calcium score; *p*^*75*^ 75th percentile; no CACS recommended in low CV-risk group, although 4% have significant CAC [42]

Conventional risk prediction models based on traditional risk factors are commonly used in a case-finding method at the general practitioners (GPs) office, without systematic screening on a population level. In view of the limited accuracy of risk prediction models and the strong predictive value of CACS for ASCVD, screening by using CACS may improve risk prediction. However, this approach is currently not included in the cardiovascular risk management (CVRM) guideline; the focus is on the potential value of CACS to assist in decision-making regarding preventive medication around treatment thresholds for the risk factor-based risk score (Fig. 1). CACS has clearly shown its added value in risk prediction in asymptomatic individuals, at least those aged between 50 and 70 years (but maybe even a broader range) with intermediate or maybe even high CV risk based on current risk prediction models, but most guidelines do not yet recommend CACS for risk management. This is partly due to the fact that the cost-effectiveness of adding CACS to risk prediction models has not been convincingly established [23]. The Dutch CVRM guideline is currently being updated and will be published in 2024. The position of CACS as a diagnostic tool for reclassification of cardiovascular risk in primary prevention will be revised. The previous guideline (2019) stated that if CACS is available one can consider to take it into account in individuals in whom there is doubt as to whether to start preventive therapy [24]. Indeed, knowing the CACS is high increases medication adherence [25]. The new CVRM guideline advises to consider determining CACS in individuals aged 50–70 years if a discussion about whether to initiate drug treatment does not yield a decision. Importantly, nowadays nearly all Dutch hospitals offer CT for CACS evaluation, and in some regions GPs can already order a CT-CACS, without referral to a medical specialist, making CT more accessible.

### CACS screening for primary prevention

So far, there is no evidence from randomised controlled trials (RCTs) that screening strategies based on CACS improve prognosis. It is important to note that this level of evidence is also lacking for the current risk prediction models, as highlighted in the 2021 ESC Guideline on Prevention [26]. The only two RCTs, the St Francis Heart study [27], and DANCAVAS [28], have not yet shown a clear benefit for treatment according to a population-based screening approach with CACS. However, in a sub-analysis of the St Francis Heart study, individuals with a positive family history of CVD *and* CACS > 80th percentile had a 45% reduction in events in the cholesterol-lowering arm as compared with placebo [27]. The DANCAVAS study, a population-based screening study comprising 46,611 men, suggested possible benefit for a broad cardiovascular screening approach including CT, but did not reach statistical significance for the primary endpoint of all-cause mortality after a follow-up of 5.6 years (HR 0.95, 95% CI 0.90–1.00, *p* = 0.06). Importantly, the primary endpoint reached borderline significance, while the study was powered for a 10-year follow-up [28]. On the other hand, in a prespecified subgroup analysis, younger participants (65–69 years) had more benefit from screening than older individuals (> 70 years). Currently, the Dutch ROBINSCA (Risk or benefit in screening for cardiovascular diseases) trial is being conducted [29]. This trial is evaluating men aged 45–74 years and women aged 55–74 years, with at least one risk factor, comparing a CACS-based strategy (intervention arm) with usual GP care (control arm). Management in the two intervention arms is evidence based. Over 43,000 individuals were included and currently 5‑year outcomes are awaited.

### Interpretation and implication of CACS

When and how to treat or not to treat based on CACS in asymptomatic individuals for primary prevention of ASCVD is still debated. Despite the lack of evidence from major RCTs, observational data have shown that a significantly elevated CACS, ≥ 100 or ≥ 75th age, sex and ethnicity corrected percentile, causes a sufficiently high risk to consider cholesterol-lowering therapy [16, 17]. Therefore, the combined European and American CVRM guidelines advise to consider cholesterol-lowering therapy if CACS is significantly elevated and to consider withholding cholesterol-lowering therapy if CACS is 0 [26, 30].

It is unknown whether there is a threshold for CACS above which aspirin therapy should be initiated. It is currently not recommended to prescribe aspirin in primary prevention because of the increased risk of bleeding [31]. On the other hand, randomised trials on aspirin use did not take into account CACS, therefore we do not know whether the individuals who benefited from aspirin therapy in these trials might have been the ones with an increased CACS. However, incorporating CACS in an aspirin-prescription decision-making tool, based on the MESA study, suggested that individuals with CACS ≥ 100 would benefit from treatment with aspirin, regardless of their risk status according to current risk prediction models (estimated 5‑year number needed to treat: 173 for low-risk individuals; 92 for high-risk individuals and 442 for a major bleed) [32].

### Reclassification to a lower ASCVD risk based on CACS 0, and withholding preventive treatment

ASCVD risk prediction should not be viewed as a static process. Risk of ASCVD evolves over the years, not only because individuals get older, but also because risk factors evolve. Therefore, it is important to realise that when CACS is 0, it needs to be repeated in the coming years. This is in line with the 2018 ACC/AHA cholesterol guidelines, which emphasise the clinical importance of CACS 0, and state that it is reasonable to withhold cholesterol-lowering therapy and, in most individuals, reassess risk with CACS in 5 to 10 years [30]. In certain very high-risk subgroups, such as familial hypercholesterolaemia, guidelines advise to continue cholesterol-lowering therapy in patients with a CACS of 0, considering their very high lifetime risk of ASCVD [33]. On the other hand, two independent prospective cohort studies showed that individuals with familial hypercholesterolaemia and CACS 0, still had a very low 10-year event rate of 0–1.2% [20, 34]. Therefore, CACS could help increase precision in familial hypercholesterolaemia patients and could help to reduce costs related to more intensified cholesterol-lowering regimens. Finally, although multiple studies comprising in total 4248 individuals with CACS 0 followed for 3 to 10 years showed that the warranty period for the development of any CAC is 3 to 7 years [35, 36], another study showed that only 2% of individuals with CACS 0 at baseline progressed to CACS > 50 in 5 years [36]. Furthermore, only individuals with a positive, low CACS level of 1–100 at baseline progressed to a substantial risk (e.g. > 7.5%, in 10 years) for which cholesterol-lowering medication is recommended [16].

### Cost-effectiveness of CACS as part of cardiovascular risk prediction

One of the reasons why CACS currently has no basis in risk prediction has to do with the fact that the cost-effectiveness analyses in the primary prevention context have so far shown conflicting results [23, 37]. Critical factors that drove the conclusions in all these studies were the costs associated with cholesterol-lowering medication, the rating of side effects from cholesterol-lowering medications and the general desire to avoid lifelong preventive therapy. In the most recent cost-effectiveness analysis by Hong et al. [23], outcomes were similar for risk prediction based on the addition of CACS or based on the 2013 ACC/AHA guidelines [38] only. They analysed the addition of CACS in the intermediate-risk group in which only 11.4% had a CACS elevated enough to start cholesterol-lowering therapy. Although for the intermediate-risk group it did not seem cost-effective to add CACS to risk prediction models, it did result in less patients receiving preventive therapy, namely 44% of individuals considered or recommended for cholesterol-lowering therapy had CACS of 0. Therefore, as a rule-in strategy CACS does not seem to be cost-effective, but as a rule-out strategy it might be. The authors concluded that cost-effectiveness analyses should not be the only criterion for clinical decision-making, but that a shared decision-making model is similarly important for clinicians, patients and policy-makers.

## Future perspectives of CT for screening CAD and CVRM

CACS can help in a more accurate reclassification of cardiovascular risk prediction and will be used as such more often in the future. Multiple studies, comprising 25,370 asymptomatic individuals, have shown that in almost one-third of individuals recommended for cholesterol-lowering medication, risk was overestimated based on current risk prediction models, since they had CACS 0, translating into an extremely low ASCVD event rate of less than 1% in 10 years [16, 17, 39, 40]. Similarly, in a Dutch population around one-third of individuals recommended for cholesterol-lowering therapy had CACS 0 [40]. Interestingly, there were also substantial differences between men and women with almost 50% of women recommended for cholesterol-lowering therapy having CACS 0. Additional biomarkers have also been proposed for risk prediction. A prospective cohort study of almost 6000 individuals, followed for 7 years, evaluated the predictive value of multiple biomarkers such as N‑terminal fragment of prohormone B‑type natriuretic peptide (NT-proBNP), von Willebrand factor, fibrinogen, leucocyte count, homocysteine, uric acid, chronic kidney disease, peripheral artery disease, C‑reactive protein, pulse wave velocity, intima media thickness and CACS [41]. They concluded that CACS best improved risk prediction when added to the Framingham risk score, with an increase in c‑statistic of 0.05. Another biomarker to consider is lipoprotein a (Lp(a)), which has shown consistent epidemiological and genetic evidence that increased values are associated with higher ASCVD risk [42]. When Lp(a) was compared with CACS, individuals with CACS 0 and elevated Lp(a) had a similar low risk of ASCVD as individuals with CACS 0 and low Lp(a) levels [43].

### *Potential role of CCTA in estimation of cardiovascular risk in asymptomatic individuals*

Important benefits of CCTA over CACS are the possibility to characterise plaque composition and characteristics, and the opportunity to evaluate response to preventive therapy. It is unclear if CCTA is useful in the general population considered for primary prevention. Studies in symptomatic individuals have shown important prognostic value for CCTA for atherosclerosis quantification [44] but not in asymptomatic populations. This is currently being investigated in several RCTs: SCOT-HEART2 (NCT03920176; *n* = 6000), DANE-HEART (NCT05677386; *n* = 6000) and TRANSFORM (NCT06112418; *n* = 7500).

The Society of Cardiovascular Computed Tomography (SCCT) 2021 expert consensus makes the following recommendations for CCTA in asymptomatic individuals: (1) it is rarely appropriate to perform CCTA in low- and intermediate-risk populations; (2) it may be appropriate to perform CCTA in selected high-risk populations, especially if they have a high probability of substantial non-calcified plaque [45]. Examples of such selected groups are young patients with familial hypercholesterolaemia [34] and individuals with a hazardous occupation, such as pilots and individuals with a high cardiovascular risk [46]. The NATO HFM-251 Occupational Cardiology in Military Aircrew working group recommends enhanced screening with CACS alone or combined with CCTA in aircrew who are identified with increased risk using a conventional risk calculator and resting ECG. In addition, the SCCT recommends CCTA: (1) as an appropriate alternative to invasive angiography and other non-invasive tests before non-coronary surgery; (2) to exclude CAD in patients with suspected non-ischaemic cardiomyopathy; (3) as an appropriate alternative to invasive angiography for coronary allograft vasculopathy in patients after heart transplantation [45].

Although there is no conclusive evidence, there are other groups that may benefit from additional CCTA. For instance, women with preeclampsia, since according to the current Dutch guidelines they carry the highest risk for ASCVD of all female specific risk factors [47]. Furthermore, various studies have shown a higher prevalence of CACS ≥ 100 and (calcified) plaque on CCTA in athletes in comparison with less active controls. Higher CACS may not represent a higher CV risk in athletes, but absence of CAC and plaque on CCTA is always better than any CACS or coronary plaque [48].

Rapid development of AI-supported algorithms to automatically quantify plaque volumes and high-risk plaque characteristics from CCTA can help to assess important prognostic markers for future cardiovascular events [49]. In conjunction, developments in genetic risk assessment and blood omics approaches, such as proteomics or lipidomics, may enable a personalised multidimensional ‘one-stop shop’ approach in ASCVD risk prediction in the near future.

## Conclusion

CACS has a role in CVRM management of asymptomatic individuals, at least those between 50 to 70 years with intermediate ASCVD risk, based on current risk prediction models using traditional risk factors. CACS improves risk prediction, although there are conflicting results on cost-effectiveness. Current guidelines recommend to consider cholesterol-lowering therapy in case of CACS ≥ 100 or ≥ 75th percentile. The absence of CAC reflects an extremely low 10-year risk of ASCVD and could be used to withhold cholesterol-lowering therapy for 5 to 10 years, but this is subject to future research. Currently, adding CCTA may only be considered in selected high-risk asymptomatic persons.

## References

[1] Damen JA, Hooft L, Schuit E, et al. Prediction models for cardiovascular disease risk in the general population: systematic review. BMJ. 2016;353:i2416. 27184143 10.1136/bmj.i2416 PMC4868251

[2] van Giessen A, Peters J, Wilcher B, et al. Systematic Review of Health Economic Impact Evaluations of Risk Prediction Models: Stop Developing, Start Evaluating. Value Health. 2017;20:718–26. 28408017 10.1016/j.jval.2017.01.001

[3] Damen JA, Pajouheshnia R, Heus P, et al. Performance of the Framingham risk models and pooled cohort equations for predicting 10-year risk of cardiovascular disease: a systematic review and meta-analysis. BMC Med. 2019;17:109. 31189462 10.1186/s12916-019-1340-7 PMC6563379

[4] Kist JM, Vos RC, Mairuhu ATA, et al. SCORE2 cardiovascular risk prediction models in an ethnic and socioeconomic diverse population in the Netherlands: an external validation study. EClinicalMedicine. 2023;57:101862. 36864978 10.1016/j.eclinm.2023.101862 PMC9971516

[5] SCORE2 working group, ESC Cardiovascular risk collaboration. SCORE2 risk prediction algorithms: new models to estimate 10-year risk of cardiovascular disease in Europe. Eur Heart J. 2021;42:2439–54. 34120177 10.1093/eurheartj/ehab309 PMC8248998

[6] Peters SA, den Ruijter HM, Bots ML, et al. Improvements in risk stratification for the occurrence of cardiovascular disease by imaging subclinical atherosclerosis: a systematic review. Heart. 2012;98:177–84. 22095617 10.1136/heartjnl-2011-300747

[7] Ties D, van der Ende YM, Pundziute G, et al. Pre-screening to guide coronary artery calcium scoring for early identification of high-risk individuals in the general population. Eur Heart J Cardiovasc Imaging. 2022;24:27–35. 35851802 10.1093/ehjci/jeac137 PMC9762935

[8] Bell KJL, White S, Hassan O, et al. Evaluation of the Incremental Value of a Coronary Artery Calcium Score Beyond Traditional Cardiovascular Risk Assessment: A Systematic Review and Meta-analysis. JAMA Intern Med. 2022;182:634–42. 35467692 10.1001/jamainternmed.2022.1262 PMC9039826

[9] McClelland RL, Jorgensen NW, Budoff M, et al. 10-Year Coronary Heart Disease Risk Prediction Using Coronary Artery Calcium and Traditional Risk Factors: Derivation in the MESA (Multi-Ethnic Study of Atherosclerosis) With Validation in the HNR (Heinz Nixdorf Recall) Study and the DHS (Dallas Heart Study). J Am Coll Cardiol. 2015;66:1643–53. 26449133 10.1016/j.jacc.2015.08.035 PMC4603537

[10] Kerr KF. Net Reclassification Index Statistics Do Not Help Assess New Risk Models. Radiology. 2023;306:e222343. 36378029 10.1148/radiol.222343 PMC9968768

[11] Paixao AR, Ayers CR, El Sabbagh A, et al. Coronary Artery Calcium Improves Risk Classification in Younger Populations. JACC Cardiovasc Imaging. 2015;8:1285–93. 26476504 10.1016/j.jcmg.2015.06.015

[12] Baber U, Mehran R, Sartori S, et al. Prevalence, impact, and predictive value of detecting subclinical coronary and carotid atherosclerosis in asymptomatic adults: the BioImage study. J Am Coll Cardiol. 2015;65:1065–74. 25790876 10.1016/j.jacc.2015.01.017

[13] Hoffmann U, Massaro JM, D’Agostino RB Sr., et al. Cardiovascular Event Prediction and Risk Reclassification by Coronary, Aortic, and Valvular Calcification in the Framingham Heart Study. J Am Heart Assoc. 2016;5:e3144. 26903006 10.1161/JAHA.115.003144 PMC4802453

[14] Temtem M, Mendonça MI, Serrao MG, et al. Predictive improvement of adding coronary calcium score and a genetic risk score to a traditional risk model for cardiovascular event prediction. Eur J Prev Cardiol. 2024;31:709–15. 38175668 10.1093/eurjpc/zwae005

[15] Sarwar A, Shaw LJ, Shapiro MD, et al. Diagnostic and prognostic value of absence of coronary artery calcification. JACC Cardiovasc Imaging. 2009;2:675–88. 19520336 10.1016/j.jcmg.2008.12.031

[16] Budoff MJ, Young R, Burke G, et al. Ten-year association of coronary artery calcium with atherosclerotic cardiovascular disease (ASCVD) events: the multi-ethnic study of atherosclerosis (MESA). Eur Heart J. 2018;39:2401–8. 29688297 10.1093/eurheartj/ehy217 PMC6030975

[17] Valenti V, B OH, Heo R, et al. A 15-Year Warranty Period for Asymptomatic Individuals Without Coronary Artery Calcium: A Prospective Follow-Up of 9,715 Individuals. JACC Cardiovasc Imaging. 2015;8:900–9. 26189116 10.1016/j.jcmg.2015.01.025 PMC4537357

[18] Nasir K, Rubin J, Blaha MJ, et al. Interplay of coronary artery calcification and traditional risk factors for the prediction of all-cause mortality in asymptomatic individuals. Circ Cardiovasc Imaging. 2012;5:467–73. 22718782 10.1161/CIRCIMAGING.111.964528

[19] Hecht HS, Narula J. Coronary artery calcium scanning in asymptomatic patients with diabetes mellitus: a paradigm shift. J Diabetes. 2012;4:342–50. 22672574 10.1111/j.1753-0407.2012.00212.x

[20] Gallo A, de Isla PL, Charriere S, et al. The Added Value of Coronary Calcium Score in Predicting Cardiovascular Events in Familial Hypercholesterolemia. JACC Cardiovasc Imaging. 2021;14:2414–24. 34274263 10.1016/j.jcmg.2021.06.011

[21] Hecht HS, de Siqueira ME, Cham M, et al. Low- vs. standard-dose coronary artery calcium scanning. Eur Heart J Cardiovasc Imaging. 2015;16:358–63. 25381303 10.1093/ehjci/jeu218

[22] Mortensen MB, Cainzos-Achirica M, Steffensen FH, et al. Association of Coronary Plaque With Low-Density Lipoprotein Cholesterol Levels and Rates of Cardiovascular Disease Events Among Symptomatic Adults. JAMA Netw Open. 2022;5:e2148139. 35147685 10.1001/jamanetworkopen.2021.48139 PMC8837910

[23] Hong JC, Blankstein R, Shaw LJ, et al. Implications of Coronary Artery Calcium Testing for Treatment Decisions Among Statin Candidates According to the ACC/AHA Cholesterol Management Guidelines: A Cost-Effectiveness Analysis. JACC Cardiovasc Imaging. 2017;10:938–52. 28797417 10.1016/j.jcmg.2017.04.014

[24] Hoes A, van Dis I, Henstra Y, et al. Cardiovasculair risicomanagement (CVRM). Richtlijnendatabase. 2019.

[25] Venkataraman P, Huynh Q, Nicholls SJ, et al. Impact of a coronary artery calcium-guided statin treatment protocol on cardiovascular risk at 12 months: Results from a pragmatic, randomised controlled trial. Atherosclerosis. 2021;334:57–65. 34482089 10.1016/j.atherosclerosis.2021.08.002

[26] Visseren FLJ, Mach F, Smulders YM, et al. 2021 ESC Guidelines on cardiovascular disease prevention in clinical practice. Eur Heart J. 2021;42:3227–337. 34458905 10.1093/eurheartj/ehab484

[27] Mulders TA, Sivapalaratnam S, Stroes ES, Kastelein JJ, Guerci AD, Pinto-Sietsma SJ. Asymptomatic individuals with a positive family history for premature coronary artery disease and elevated coronary calcium scores benefit from statin treatment: a post hoc analysis from the St. Francis Heart Study. JACC Cardiovasc Imaging. 2012;5:252–60. 22421169 10.1016/j.jcmg.2011.11.014

[28] Lindholt JS, Sogaard R, Rasmussen LM, Mejldal A, Lambrechtsen J, Steffensen FH, et al. Five-Year Outcomes of the Danish Cardiovascular Screening (DANCAVAS) Trial. N Engl J Med. 2022;387:1385–94. 36027560 10.1056/NEJMoa2208681

[29] van der Aalst CM, Denissen S, Vonder M, et al. Screening for cardiovascular disease risk using traditional risk factor assessment or coronary artery calcium scoring: the ROBINSCA trial. Eur Heart J Cardiovasc Imaging. 2020;21:1216–24. 32584979 10.1093/ehjci/jeaa168

[30] Grundy SM, Stone NJ, Bailey AL, et al. AHA/ACC/AACVPR/AAPA/ABC/ACPM/ADA/AGS/APhA/ASPC/NLA/PCNA Guideline on the Management of Blood Cholesterol: A Report of the American College of Cardiology/American Heart Association Task Force on Clinical Practice Guidelines. J Am Coll Cardiol. 2018;2019(73):e285–e350. 10.1016/j.jacc.2018.11.003 30423393

[31] Berger JS, Lala A, Krantz MJ, et al. Aspirin for the prevention of cardiovascular events in patients without clinical cardiovascular disease: a meta-analysis of randomized trials. Am Heart J. 2011;162(e2):115–24. 21742097 10.1016/j.ahj.2011.04.006

[32] Miedema MD, Duprez DA, Misialek JR, et al. Use of coronary artery calcium testing to guide aspirin utilization for primary prevention: estimates from the multi-ethnic study of atherosclerosis. Circ Cardiovasc Qual Outcomes. 2014;7:453–60. 24803472 10.1161/CIRCOUTCOMES.113.000690 PMC4412344

[33] Ibrahim S, Reeskamp LF, de Goeij JN, et al. Beyond Early LDL Cholesterol Lowering to Prevent Coronary Atherosclerosis in Familial Hypercholesterolemia. Eur J Prev Cardiol. 2024;31:892–900. 38243822 10.1093/eurjpc/zwae028

[34] Miname MH, Bittencourt MS, Moraes SR, et al. Coronary Artery Calcium and Cardiovascular Events in Patients With Familial Hypercholesterolemia Receiving Standard Lipid-Lowering Therapy. JACC Cardiovasc Imaging. 2019;12:1797–804. 30448145 10.1016/j.jcmg.2018.09.019

[35] Gopal A, Nasir K, Liu ST, et al. Coronary calcium progression rates with a zero initial score by electron beam tomography. Int J Cardiol. 2007;117:227–31. 16875746 10.1016/j.ijcard.2006.04.081

[36] Dzaye O, Dardari ZA, Cainzos-Achirica M, et al. Warranty Period of a Calcium Score of Zero: Comprehensive Analysis From MESA. JACC Cardiovasc Imaging. 2021;14:990–1002. 33129734 10.1016/j.jcmg.2020.06.048 PMC8076346

[37] van Kempen BJ, Ferket BS, Steyerberg EW, et al. Comparing the cost-effectiveness of four novel risk markers for screening asymptomatic individuals to prevent cardiovascular disease (CVD) in the US population. Int J Cardiol. 2016;203:422–31. 26547049 10.1016/j.ijcard.2015.10.171

[38] Goff DC Jr., Lloyd-Jones DM, Bennett G, et al. ACC/AHA guideline on the assessment of cardiovascular risk: a report of the American College of Cardiology/American Heart Association Task Force on Practice Guidelines. J Am Coll Cardiol. 2013;63(25):2935–59. 24239921 10.1016/j.jacc.2013.11.005 PMC4700825

[39] Nasir K, Bittencourt MS, Blaha MJ, et al. Implications of Coronary Artery Calcium Testing Among Statin Candidates According to American College of Cardiology/American Heart Association Cholesterol Management Guidelines: MESA (Multi-Ethnic Study of Atherosclerosis). J Am Coll Cardiol. 2015;66:1657–68. 26449135 10.1016/j.jacc.2015.07.066

[40] Xia C, Vonder M, Sidorenkov G, et al. Cardiovascular Risk Factors and Coronary Calcification in a Middle-aged Dutch Population: The ImaLife Study. J Thorac Imaging. 2021;36:174–80. 33060489 10.1097/RTI.0000000000000566 PMC8132906

[41] Kavousi M, Elias-Smale S, Rutten JH, et al. Evaluation of newer risk markers for coronary heart disease risk classification: a cohort study. Ann Intern Med. 2012;156:438–44. 22431676 10.7326/0003-4819-156-6-201203200-00006

[42] Tsimikas S, Fazio S, Ferdinand KC, et al. NHLBI Working Group Recommendations to Reduce Lipoprotein(a)-Mediated Risk of Cardiovascular Disease and Aortic Stenosis. J Am Coll Cardiol. 2018;71:177–92. 29325642 10.1016/j.jacc.2017.11.014 PMC5868960

[43] Mehta A, Vasquez N, Ayers CR, et al. Independent Association of Lipoprotein(a) and Coronary Artery Calcification With Atherosclerotic Cardiovascular Risk. J Am Coll Cardiol. 2022;79:757–68. 35210030 10.1016/j.jacc.2021.11.058 PMC10966924

[44] Williams MC, Kwiecinski J, Doris M, et al. Low-Attenuation Noncalcified Plaque on Coronary Computed Tomography Angiography Predicts Myocardial Infarction: Results From the Multicenter SCOT-HEART Trial (Scottish Computed Tomography of the HEART). Circulation. 2020;141:1452–62. 32174130 10.1161/CIRCULATIONAHA.119.044720 PMC7195857

[45] Narula J, Chandrashekhar Y, Ahmadi A, et al. SCCT 2021 Expert Consensus Document on Coronary Computed Tomographic Angiography: A Report of the Society of Cardiovascular Computed Tomography. J Cardiovasc Comput Tomogr. 2021;15:192–217. 33303384 10.1016/j.jcct.2020.11.001 PMC8713482

[46] Gray G, Davenport ED, Bron D, et al. The challenge of asymptomatic coronary artery disease in aircrew; detecting plaque before the accident. Heart. 2019;105(Suppl 1):s17–s24. 30425082 10.1136/heartjnl-2018-313053 PMC6256297

[47] Duvekot JJ, Maas AHEM, Roeters van Lennep JE, et al. CVRM na een reproductieve aandoening. Richtlijnendatabase. 2023.

[48] Aengevaeren VL, Mosterd A, Sharma S, et al. Exercise and Coronary Atherosclerosis: Observations, Explanations, Relevance, and Clinical Management. Circulation. 2020;141:1338–50. 32310695 10.1161/CIRCULATIONAHA.119.044467 PMC7176353

[49] Nurmohamed NS, Bom MJ, Jukema RA, et al. AI-Guided Quantitative Plaque Staging Predicts Long-Term Cardiovascular Outcomes in Patients at Risk for Atherosclerotic CVD. Jacc: Cardiovasc Imaging. 2024;17:269–80. 37480907 10.1016/j.jcmg.2023.05.020

